# Neutral Dietary Effects of Two MicroRNAs, Csu-Novel-260 and Csu-Mir-14, on the Non-Target Arthropod *Folsomia candida*

**DOI:** 10.3390/plants12091885

**Published:** 2023-05-05

**Authors:** Qinli Zhou, Lanzhi Han, Yunhe Li, Jing Li, Xiaowei Yang

**Affiliations:** 1 College of Plant Protection, Hebei Agricultural University, Baoding 071001, China; 2 State Key Laboratory for Biology of Plant Diseases and Insect Pests, Institute of Plant Protection, Chinese Academy of Agricultural Sciences, Beijing 100193, China; 3 State Key Laboratory of Cotton Bio-Breeding and Integrated Utilization, School of Life Sciences and College of Agriculture, Henan University, Kaifeng 475004, China

**Keywords:** microRNA, non-target arthropod, risk assessment, *Folsomia candida*, genetically engineered crops

## Abstract

RNA interference (RNAi) that is triggered by small or short RNAs has shown enormous potential in the development of pest control strategies. Two microRNAs (miRNAs), Csu-novel-260 and Csu-miR-14, were used in insect-resistant genetically engineered (IRGE) rice lines to confer resistance to *Chilo suppressalis*. However, a risk assessment of RNAi-based products is essential to determine the safety of a biopesticide or IRGE crop for commercialization. The non-target organism *Folsomia candida*, which plays an important ecological role as a soil decomposer in agricultural ecosystems, was used to assess the risk of miRNAs Csu-novel-260 and Csu-miR-14. In this study, a dietary miRNA toxicity assay system was established in *F. candida*. The expression levels of target genes, survival rate, fecundity and body size were investigated to evaluate the effects of the miRNAs on *F. candida* under the worst-case scenario. The results showed that the dietary miRNA toxicity assay system could be used for risk assessment of miRNA in *F. candida*. The target genes of miRNAs were influenced by miRNA at some time points. However, no significant differences were observed in the life-table parameters in *F. candida* fed with a diet containing miRNAs. The dietary effects of two miRNAs on *F. candida* are neutral.

## 1. Introduction

RNA interference (RNAi) that is triggered by small or short RNAs has shown enormous potential in the development of pest control strategies [1,2,3]. It can effectively and specifically silence target genes, resulting in mortality or disrupting development [4]. RNAi-based biocontrol products have already been used to trigger gene silencing in insects for pest control, including biopesticide and insect-resistant genetically engineered (IRGE) crops that produce double-stranded RNA (dsRNA), hairpin RNAs or microRNAs (miRNAs) [1]. For instance, in 2007, two RNAi-based IRGE crops were reported; one of the crops was a corn that expressed dsRNA against the western corn rootworm (*Diabrotica virgifera virgifera*) [5], while the other crop produced hairpin RNAs that targeted the cotton bollworm (*Helicoverpa armigera*) [6]. Additionally, IRGE rice producing miRNA Csu-novel-260 [7] or Csu-miR-14 [8] were found to be resistant to striped stem borer (*Chilo suppressalis*) under field conditions.

However, the potential to use the technology for pest control has led to concern about the ecological risk assessment of RNAi-based products, with a particular focus on the effects on non-target organisms that represent diverse ecological functions, including pollinators, soil decomposers and natural enemies [9,10,11,12]. A substantial body of literature has been published reporting studies on the effects of genetically modified plants on non-target arthropods. Most of the studies show neutral or “negligible” effects, while some reported negative or positive effects [13]. For example, studies have shown that dsRNA targeting the v-ATPase A gene of western corn rootworm has negligible effects on larvae and adults of honeybee (*Apis mellifera*) [14], monarch larvae (*Danaus plexippus*) [15] and collembolan (*Sinella curviseta*) [16]. However, several ladybird species have shown differential responses [17,18].

Collembolans play an important ecological role as consumers of plant residues and soil fungi in agricultural ecosystems, and they might be exposed to RNAi-based products [19]. A common species, *Folsomia candida*, has a long history of being used as a “standard” test organism for estimating the effect of pesticides and environmental pollutants on non-target soil arthropods since it is parthenogenetic and is easy to maintain in the laboratory [20]. *F. candida* has also been widely used for assessing the non-target effects of insecticide and IRGE crops [21,22]. Up to now, most of these studies have been performed on *Bacillus thuringiensis* (Bt) crops [22,23,24,25]. A few studies have also been carried out on RNAi-based IRGE crops. For example, DvSSJ1 dsRNA expressed by maize is not expected to be harmful to *F. candida* populations [26]. *F. candida* was not negatively impacted when exposed to dsRNA targeting western corn rootworm [27]. To our knowledge, the risk assessment of miRNAs in *F. candida* has not been reported.

A tiered approach to risk assessment of transgenic crops was used internationally [28,29]. Diets containing Bt protein or dsRNA were usually used in first-step laboratory studies aimed at analyzing toxic effects on non-target organisms. However, there is a lack of studies on the risk assessment of miRNAs, and no consensus has been reached on whether they should be included due to the possible unintended effects on transgenic plants. Nonetheless, dietary exposure studies are effective initial steps in evaluating the environmental risks of RNAi-based biocontrol products on non-target organisms. The dietary miRNA approach is expected to introduce concentrations of testing compounds 10–100 times or more than those found in plants to non-target organisms, and it is cost-effective and time-saving. In this study, a first-tier (laboratory-scale) experiment system using an artificial diet was developed to expose *F. candida* to high doses of miRNAs, with the aim of determinizing their potential effects on the non-target organism *F. candida*. A suitable positive control was identified, and the stability of miRNA in the artificial diet, as well as the uptake of miRNA by *F. candida*, was checked. The effects of two miRNAs (Csu-novel-260 and Csu-miR-14) that have shown promise in controlling *C. suppressalis* on *F. candida* were investigated in the first-tier experiments. The binding probability of miRNA and homologs of target genes were predicted in this study. The expression levels of target genes, survival rate, fecundity and body size were investigated to evaluate the effects of the miRNAs on *F. candida* under the worst-case scenario. It should be noted that a previous study reported neutral effects of miRNA Csu-novel-260 on the non-target organism *A. mellifera* [30].

## 2. Results

### 2.1. Binding Probability of miRNA and Target Genes

**Figure 1:**
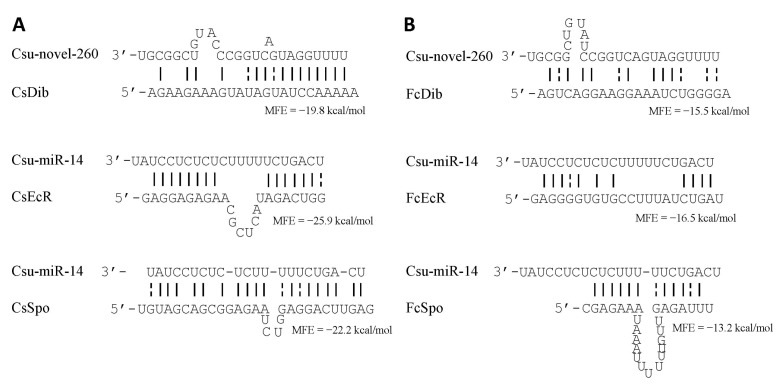
Binding probability of Csu-novel-260 and Csu-miR-14 with their target genes in *C. suppressalis* (**A**) and *F. candida* (**B**). Cs refers to *C. suppressalis*; Fc refers to *F. candida. CsDib* is the target gene of Csu-novel-260, while *CsEcR* and *CsSpo* are the target genes of Csu-miR-14 in *C. suppressalis.* Alignment and the MFE between miRNA and UTRs were predicted by RNAhybrid.

Disembodied (*Dib*) is target gene of Csu-novel-260, while ecdysone receptor (*EcR*) and spook (*Spo*) are target genes of Csu-miR-14 in *C. suppressalis* (NCBI accession numbers: KX833964.1, AB067811.1 and MN010764.1) [7,8]. The homologous genes of these target genes in *F. candida* were identified by sequence similarity (NCBI accession numbers: XM_035854100.1, XM_035846430.1 and XM_022094060.2) and confirmed by Sanger sequencing. No potential binding sites were found between Csu-novel-260 and *F. candida Dib* (*FcDib*) gene or between Csu-miR-14 and *F. candida Spo*/*EcR* (*FcSpo*/*FcEcR*) genes by miRanda even with a low threshold minimum free energy (MFE) at −1 kcal/mol. RNAHybrid shows that there are potential binding sites with a low probability between the miRNAs and their target genes. The MFEs are −15.5 kcal/mol, −16.5 kcal/mol and −13.2 kcal/mol, respectively (Figure 1). These results suggest that there is a small possibility that both miRNAs interact with the target genes in *F. candida* and induce mRNA degradation. 

### 2.2. Response of F. candida to Chlorpyrifos

**Figure 2:**
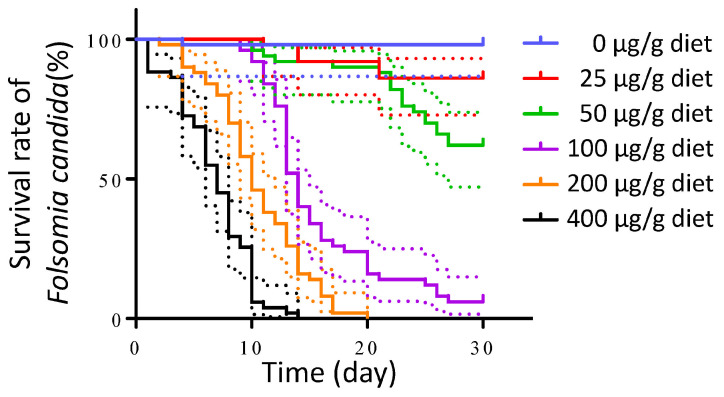
Survival of *F. candida* after feeding on artificial diets containing different concentrations of chlorpyrifos. The Kaplan–Meier survival curves were created using GraphPad Prism. Dotted line represents the 95% confidence interval (CI).

Chlorpyrifos (CPF) was chosen as the positive control to evaluate the test validity of the experimental procedure based on a preliminary experiment. The mortality rate of *F. candida* increased with the CPF concentration in the artificial diets. Kaplan–Meier survival curves were generated (Figure 2), and a log-rank (Mantel–Cox) test indicated significant differences in the survival rate of *F. candida* among the artificial diets with varying CPF concentrations (df = 5, *p* < 0.0001). At CPF concentrations below 50 μg/g diet, the mortality after 30 days remained 38%. However, when the CPF concentration reached 400 μg/g diet, all test insects died at 14 days. An appropriate concentration of the positive control should be high enough to ensure that the mortality is easy to observe. The concentration should not be too high to prevent all the insects from dying within a few days since the experiment spans 30 days. At 100 or 200 μg/g diet, the survival rate was 0% at 20 days or 0.6% at 30 days (Figure 2). Therefore, a concentration range of 100–200 μg/g diet was deemed appropriate for this assay system, and 200 μg/g diet was used in the subsequent experiment.

### 2.3. Stability of miRNA in Artificial Diet

**Figure 3:**
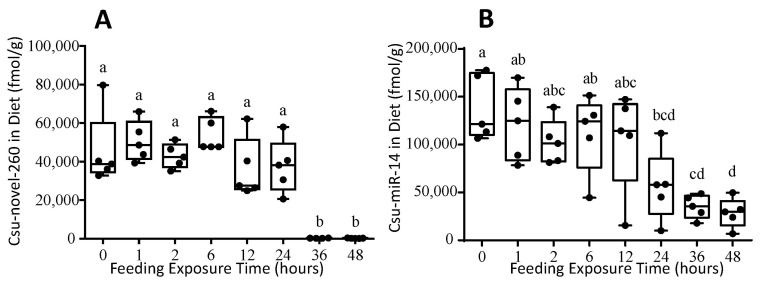
The stability of miRNA Csu-novel-260 (**A**) and Csu-miR-14 (**B**) in artificial diet for *F. candida*. The diet was placed in the experiment environment and exposed to springtail feeding. The data are shown as means ± SE, and each dot presents a biological replicate, *n* = 5. Different letters on the bar indicate significant differences among different time points (Tukey HSD test, *p* < 0.05).

The miRNA was diluted in RNase-free water and added to baker’s yeast. Initially, 233,700 fmol miRNA was added per gram dry diet. After dilution, lyophilization and grinding, only 45,547 fmol/g diet of Csu-novel-260 and 138,234 fmol/g diet of Csu-miR-14 remained in the artificial diet. The miRNA concentration decreased slowly in the first 24 h of feeding exposure. The concentrations of two miRNAs in the artificial diet were 37,618 fmol/g and 56,758 fmol/g at 24 h. Then they quickly decreased to 281 fmol/g and 35,163 mol/g only at 36 h, and they decreased to even lower values at 48 h (Figure 3). These results suggest that miRNAs in yeast powder were degraded soon after 24 h. It is suggested that the artificial diet containing miRNA be replaced every 24 h. 

### 2.4. Uptake of miRNA in F. candida

**Figure 4:**
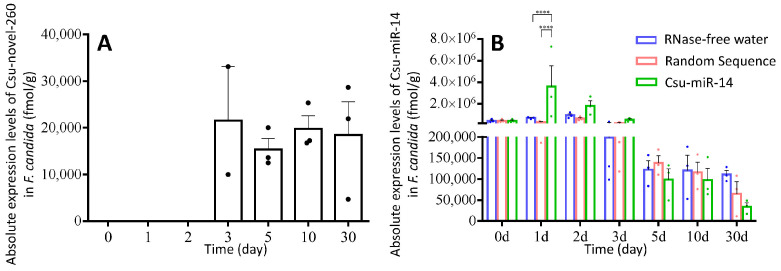
(**A**) The absolute expression levels of Csu-novel-260 in *F. candida* with Csu-novel-260 treatment. (**B**) The absolute expression levels of Csu-miR-14 in *F. candida* with different treatments. No Csu-novel-260 was detected in RNase-free water and random sequence treatments. Csu-miR-14 could be detected in control collembolans and varied during the experiment period. The data are shown as means  ±  SE, and each dot presents a biological replicate, *n* = 3. One-way ANOVA was used for the analysis of Csu-novel-260 (F_3,7_ = 0.190, *p* = 0.900). Two-way ANOVA was used for the analysis of Csu-miR-14 (Time: F_6,42_ = 5.149, *p* = 0.0005; Treatment: F_2,42_ = 4.839, *p* = 0.0128; Interaction: F_12,42_ = 2.396, *p* = 0.0183). Asterisks indicate significant differences (**** *p* < 0.0001).

The absolute expression levels of two miRNAs were measured when *F. candida* was fed a diet containing Csu-novel-260 or Csu-miR-14 (Figure 4). Csu-novel-260 was not detected in RNase-free water-treated and random sequence-treated collembolans, and it was not detected in the first two days of Csu-novel-260-treated collembolans. The concentrations of Csu-novel-260 detected at 3 days, 5 days, 10 days and 30 days were 21,562 ± 11,542, 15,381 ± 2340, 19,798 ± 2788 and 18,442 ± 7136 fmol/g weight. Csu-miR-14 was detected in untreated collembolans and was supposed to exist in *F. candida* (Figure 4B). When the springtails were fed the diet containing Csu-miR-14, the miRNA detected in their bodies was significantly affected by the treatment (Figure 4B, two-way ANOVA, factor treatment, F_2,42_ = 4.839, *p* = 0.0128). These results suggest that collembolans can uptake miRNAs through an artificial diet and keep them in vivo.

### 2.5. The Expression Levels of Target Genes of miRNA

**Figure 5:**
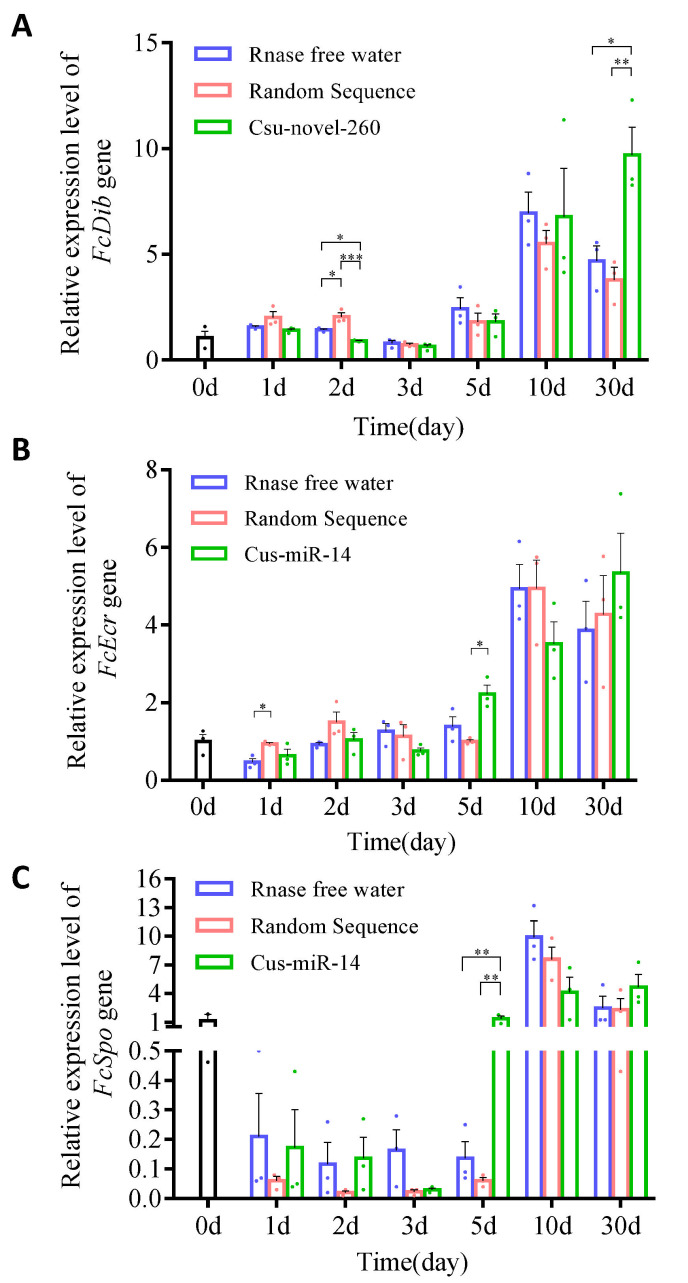
The relative expression levels of three target genes of Csu-novel-260 and Csu-miR-14. The genes are (**A**) *FcDib*, (**B**) *FcEcR* and (**C**) *FcSpo.* The expression levels of the genes in all the samples were normalized to their expression levels in untreated groups at 0 days. The data are shown as mean ± SE, and each dot presents a biological replicate, *n* = 3. The significant differences between different treatment groups are shown by asterisks (* *p* < 0.05, ** *p* < 0.01, *** *p* < 0.001).

The relative expression levels of three target genes (*FcDib*, *FcEcR* and *FcSpo*) were measured during the 30-day experiment and compared among three treatment groups: RNase-free water, random sequence treatment, and treatment with Csu-novel-260 or Csu-miR-14. The expression levels of these genes varied during the 30 days in the control group, with *FcDib* showing the most variation from 0.81 to 6.95 (Figure 5).

During the first five days, the expression levels of three genes were affected by Csu-novel-260 or Csu-miR-14 treatment on one or two of the five days, but *FcEcR* and *FcSpo* quickly recovered to the same level as the control group, while the *FcDib* gene did not. The expression level of *FcDib* was significantly increased after the collembolans were fed a diet containing Csu-novel-260 for 30 days, as determined by a two-way ANOVA analysis with treatment and time factors. The analysis showed a significant effect of treatment (F_2,6_ = 12.000, *p* = 0.008) on the expression levels of *FcDib* (Figure 5). These results suggest that miRNA uptake by collembolans can affect the expression levels of their target genes, but the effects are mostly transient and not significant under most conditions. 

### 2.6. Effects on Life-Table Parameters

**Figure 6:**
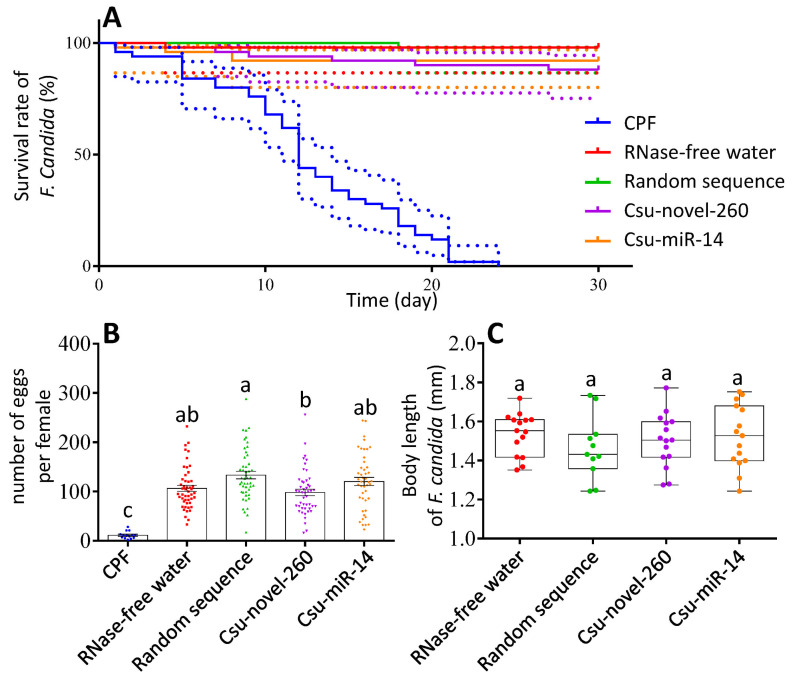
miRNA effects on life-table parameters in *F. candida*. (**A**) Survival rate of *F. candida* in the bioassay. (**B**) Number of eggs per female in the bioassay. (**C**) Body length of *F. candida* in the bioassay. CPF was used as PC. The data are shown as the mean ± SE, and each dot presents a biological replicate, *n* = 5. Log-rank (Mantel–Cox) test shows the survival rate of *F. candida* in positive control is significantly different from that in other groups (df = 4, *p* < 0.0001). There are no differences between other test groups. Dotted line represents the 95% CI. Different letters on the bars represent significant differences among different treatments (Tukey HSD test, *p* < 0.05).

The effects of miRNAs on life-table parameters were analyzed. Results showed that the survival rate of *F. candida* was not affected by miRNAs (Figure 6A). However, the survival rate of *F. candida* in the CPF-treated group (PC) was significantly different from that of other treatment groups (Figure 6A). The survival rate of *F. candida* in the other four groups, namely the Csu-novel-260-treated group, Csu-miR-14-treated group, RNase-free water-treated group (CK) and random sequence-treated group (NC), was consistently high, ranging from 88 to 98%. No significant differences were observed among these four groups.

The number of eggs produced per female and body size were analyzed (Figure 6B,C). The CPF group had a significantly lower number of eggs per female compared to the other groups, likely due to the high mortality rate during the experiment. We observed a significant difference in the number of eggs per female between the Csu-novel-260 group and the NC group, but not between the Csu-novel-260 group and the CK group (Figure 6B). Body length did not show any significant differences among the four groups, except for the CPF group (Figure 6C).

## 3. Discussion

One of the major concerns associated with RNAi-based products is their potential effects on non-target arthropods within the agricultural ecosystem [10]. Therefore, it is essential to evaluate these effects on non-target organisms before commercialization. According to a guideline of environmental safety standard, Tier I assays are recommended under the worst-case scenarios in the laboratory [31,32]. A previous study demonstrated the non-target impacts of miRNA Csu-novel-260 on *A. mellifera* [30]. In this study, miRNAs Csu-novel-260 and Csu-miR-14, which show high resistance to rice stem borer and have the potential for pest control [7,33], were assessed in *F. candida* and showed neutral effects on collembolans. 

Prior to conducting the toxicity assay, the probability of miRNAs binding to target genes in *F. candida* was analyzed. The results revealed that Csu-novel-260 and Csu-miR-14 have the potential to bind to the 3′ UTR of homologs of target genes of miRNA in *F. candida*. However, it should be noted that the structures of the binding complex are not stable, as indicated by the high MFE value (Figure 1). It is also important to note that there may be more potential binding sites in *F. candida*, particularly miRNA binding to the target RNA through partial complementarity [34].

The dietary exposure assay in *F. candida* has been previously used for Bt protein, but not for miRNA [23]. The uptake mechanism and low stability of miRNA differ from those of Bt protein. Therefore, the existing framework for assessing the safety of Bt crops should be optimized to accommodate the unique characteristics of RNAi-based products [31,35]. To ensure that the dietary exposure conditions are suitable for testing the potential harmful effects of miRNAs on *F. candida*, RNase-free water and CPF are tested as CK and PC, respectively, in this assay system (Figure 2). The survival rate of *F. candida* was 98% in the control during the 30-day experiment. The survival rate for CPF was dose-dependent, and 100–200 μg/g diet was deemed suitable for the assay. The miRNA in the artificial diet was observed to be relatively stable over a 24 h period (Figure 3) and recommended to be replaced daily to ensure that the miRNA concentration remained 161–592 times higher than the expression level in the IRGE rice [36]. In this study, the initial concentration of miRNA in the artificial diet was 1000 times higher than that in terminal leaves of a transgenic rice line. These findings provide critical information that this dietary exposure assay system is suitable for detecting the potential detrimental effects of miRNAs.

The uptake of miRNAs into the collembolans was confirmed by the absolute expression level of the miRNAs, and their expression level changes in vivo were shown (Figure 4). It is possible that the miRNAs can be packaged into extracellular vesicles or absorbed by the gut epithelium and enter the hemolymph directly in *F. candida*. However, the specific mechanisms by which miRNAs are able to survive in the insect and enter the hemolymph are still not fully understood. We concluded that the Csu-novel-260 does not exist in *F. candida*, while Csu-miR-14/miR-14 exists. It has been reported that miR-14 is present in various insects, such as *Drosophila* [37], *Bombyx mori* [38] and *A. mellifera* [39]. The target genes of miR-14 are involved in ecdysone signaling in many insects [8,38,40,41]. When the collembolans were fed with Csu-novel-260, the expression level of *FcDib* was approximately 2 times higher than that in CK and NC groups at 30 days (Figure 5A), and the number of eggs produced per female was reduced by 26.53% compared to that in the NC groups, but there was no significant difference compared to that in the CK group. No significant difference in the number of eggs was observed between CK and NC groups either. Because the concentration of Csu-novel-260 was several hundred times higher than that in fresh plant tissue and the miRNA is unstable under natural conditions, no significant differences were observed between the Csu-novel-260-treated group and RNase-free water-treated group. In conclusion, Csu-novel-260 is unlikely to pose a threat to the population size of springtails under field conditions. Similarly, Csu-miR-14 showed neutral dietary effects on *F. candida*.

Collembolans are important decomposers in soil, with a unique ecological function. Collembolans, particularly *F. candida*, have been the focus of attention for the environmental risk assessment of IRGE crops [22,23,25]. Many previous studies demonstrated the negative impacts of Bt toxin or dsRNA on collembola [16,24,26,42]. However, a few studies revealed that Bt corn caused a significant negative effect on collembola [43,44]. A long-term “minor” effect of Csu-novel-260 was shown on the expression level of the *FcDib* gene and the number of eggs produced by females, although it is not likely to be recurrent under field conditions. Therefore, it is essential to evaluate miRNAs on non-target arthropods before their commercial use, even if the miRNA is supposedly highly specific.

While Bt crops have achieved great success in the past, the sporadic emergence of resistance in target insects and limitations on target pest species have prompted researchers to explore new technological approaches [45,46]. In recent years, the use of RNAi has emerged as a promising alternative to the Bt protein-based approach for the development of IRGE crops [46]. The first RNAi-based IRGE maize was approved for commercial use in 2017 [47]. It should be noted that most studies on RNAi-based insect resistance have been based on dsRNA. However, the delivery efficiency of dsRNA is often low, making it difficult to produce enough stable dsRNA [48], and dsRNA may be ineffective in some pests [49,50]. miRNA showed a better delivery and uptake efficiency for RNAi-based IRGE cops [48,49].

In summary, a dietary miRNA toxicity assay system was established to assess the potential effects of miRNAs on *F. candida*, and two miRNAs were evaluated for their risk. The results suggest that the potential effects of miRNAs on *F. candida* are neutral, supporting the use of miRNA-based approaches for pest control in agriculture. However, there are a few considerations to take into account. Firstly, the actual exposure of *F. candida* to miRNAs in the plant and in the field can be more complicated and may require further theoretical or experimental support. Secondly, it is important to conduct risk assessments on other representative non-target species before commercialization.

## 4. Materials and Methods

### 4.1. Insect Strain and Rearing

The FCDK/Berlin strain of the soil collembolan *F. candida* [23] was obtained from the Shanghai Institute of Biological Sciences; it originally came from the former Department of Terrestrial Ecology at Aarhus University. The cultures were reared in Petri dishes (diameter 90 mm; height 10 mm), which were filled with plaster of Paris, activated charcoal and distilled water in a ratio of 8:1:7 (*w*:*w*:*w*) to cover the bottom (referred to as plaster–charcoal base or dish below). The dishes were kept at 20 °C in total darkness, and the relative humidity was ~80%. Baker’s yeast (AB Mauri Food Sales & Marketing Co., Ltd., Beijing, China) was provided as food every week at the top center of the plaster–charcoal base. 

Fifty collembolans were placed in one plaster–charcoal dish with baker’s yeast to obtain the eggs. After 48 h, the adult springtails and residual yeast were removed. Distilled water was supplied to keep the plaster–charcoal base moist. The eggs hatched after 7–8 days, and the neonates were fed with untreated yeast powder if they were not immediately used for the experiments. Collembolans that were 12 days old were used for the following experiments. 

### 4.2. miRNAs and Testing Compounds

The agomirs used in the experiment were Csu-novel-260 (TTTTGGATGACTGGCCCATGTCGGCGT), Csu-miR-14 (TCAGTCTTTTTCTCTCTCCTAT), and a random shuffled sequence of Csu-novel-260. In this experiment, miRNA agomirs and reagent CPF (Sigma-Aldrich, St. Louis, MO, USA) were tested. They were mixed with baker’s yeast, and the required amounts of agomirs or CPF were diluted in 10 mL of RNase-free water and mixed with 5 g of yeast powder. The mixture was then lyophilized and ground into powder again. The diet was kept at −80 °C until it was fed to *F. candida*. 

The miRNA agomir was a chemically modified, cholesterylated, stable miRNA mimic. The agomirs used in this experiment were commercially synthesized by Sangon Biotech (Shanghai, China). The RNase-free water was used as the blank control (CK), the random sequence supplied was used as the negative control (NC) and CPF was used as the positive control (PC). The concentration of miRNA was determined as 233,700 fmol/g dry diet, which is 1000 times the concentration of Csu-novel-260 in the terminal leaves of transgenic rice, which have the highest expression level among all tested tissues according to a previous study [36].

### 4.3. Binding Probability of miRNA and Target Genes in F. candida

The target genes of miRNA Csu-novel-260 were *Dib* gene and *Spo/EcR*, respectively, in *C. suppressalis*. To analyze the binding probability between the miRNAs and the homologs of these three target genes in *F. candida*, the programs miRanda (https://cbio.mskcc.org/miRNA2003/miranda.html, accessed on 17 March 2023) [51] and RNAhybrid (https://bibiserv.cebitec.uni-bielefeld.de/rnahybrid/welcome.html, accessed on 17 March 2023) [52] were used with the highest sensitivity parameters. The sequences of *FcDib*, *FcSpo* and *FcEcR* were obtained from NCBI (accession numbers: XM_035854100.1, XM_035846430.1 and XM_022094060.2) and verified by Sanger sequencing.

### 4.4. Determination of Positive Control

CPF was selected based on a preliminary experiment, and different concentrations were tested in *F. candida*. The baker’s yeast with different concentrations of CPF water solution (0, 25, 50, 100, 200, 400 μg/g) was placed in the collembolan rearing system. To set up the experiment, a 12-day-old collembolan was placed in a 30 mL polystyrene plastic cup (height 4 cm, diameter 4.4 cm at the top and 3 cm at the bottom). A total of 50 replicates were set up for each concentration, and the diet was replaced every day. If necessary, distilled water was added to keep the substrate humidity. The survival of each springtail was observed every day. 

### 4.5. Stability of miRNA in Artificial Diet

The miRNA agomirs were added to the yeast following the protocol described above, and the initial concentration was set as 233,700 fmol/g dry diet. The yeast powder was then placed in the center of the moistened plaster–charcoal base. Ten 12-day-old collembolans were introduced to each dish. Three replicates were set up for each miRNA, and samples of the yeast powder were collected at eight time points (0 h, 1 h, 2 h, 6 h, 12 h, 24 h, 36 h, 48 h) and weighed. The samples were stored at −80 °C until miRNA extraction for qPCR analysis to determine the stability of the miRNAs over time in the artificial diet.

### 4.6. Uptake of miRNA and Expression Level of Target Genes in F. candida

Around 200 12-day-old collembolans were collected in each replicate and placed in a plaster–charcoal dish. Four yeast powders were provided for diet as four treatments. The yeast powders were mixed with RNase-free water, Csu-novel-260 agomir, Csu-miR-14 agomir or random sequence agomir; lyophilized; and ground into powder. The initial concentration of agomir was 233,700 fmol/g dry diet. The diets were replaced every day. The insects were collected at seven time points: 0 days, 1 days, 2 days, 3 days, 5 days, 10 days and 30 days; approximately 100 individuals at 0–3 days and 20 individuals at 5 days, 10 days and 30 days were collected for each replicate. Three replicates were set and sampled at each time point. The samples were stored at −80 °C until use.

### 4.7. miRNA Effects on Life-Table Parameters

Twelve-day-old collembolans were subjected to five different treatments: Csu-novel-260 agomir, Csu-miR-14 agomir, random shuffled sequence of Csu-novel-260, CPF and RNase-free water. The agomirs were administered at an initial concentration of 233,700 fmol/g diet, while CPF was administered at a concentration of 200 μg/g diet as determined in the previous section. Individual collembolans were placed in 30 mL polystyrene plastic cups (height 4 cm, diameter 4.4 cm at the top and 3 cm at the bottom). A total of 50 replicates were set up for each treatment. The survival and egg-laying of each collembolan were monitored and recorded daily. At the end of the experiment, three springtails were randomly selected from each replicate, and their body length was measured.

### 4.8. Real-Time Quantitative PCR

miRNA in the artificial diet was isolated using TRIzol (Invitrogen, Waltham, MA, USA), while total RNA containing small RNA in *F. candida* was extracted using the miRcute microRNA isolation kit (Tiangen, Beijing, China) according to the manufacturer’s protocol for total RNA extraction. For the miRNA assay, 1 μg of miRNA from diet or total RNA from insects was reverse transcribed using the miRNA 1st Strand cDNA Synthesis Kit (by stem-loop) (Vazyme Biotech, Nanjing, Jiangsu, China) following the manufacturer’s protocol. The specific stem-loop primers (Bulge-Loop Csu-novel-260 stem-loop Primer and Bulge-Loop Csu-miR-14 stem-loop Primer) (Table 1) for reverse transcription were synthesized by Guangzhou RiboBio Co., Ltd. (Guangzhou, Guangdong, China). The miRNA expression levels were detected by a real-time quantitative PCR assay (qPCR) using miRNA Universal SYBR qPCR Master Mix (Vazyme Biotech, Nanjing, Jiangsu, China). A standard curve was generated using pure Csu-novel-260 agomir. The absolute miRNA expression levels were calculated using the standard curve method.

In the mRNA assay, 1 μg of total RNA was reverse transcribed using TransScript One-Step gDNA Removal and cDNA Synthesis SuperMix kit (TransGen, Beijing, China). The qPCR was performed using PerfectStart Green qPCR SuperMix (TransGen, Beijing, China). The relative expression levels of each gene were calculated using the 2−ΔΔCT method. The *SDHA* gene was used as the internal control [53]. All the primers used for qPCR were listed in Table 1.

### 4.9. Statistical Analysis

Log-rank (Mantel–Cox) test was used for survival data. For comparison of differences among groups under different conditions such as stability of miRNA in diet, gene expression levels and number of eggs, one-way ANOVA followed by Tukey’s HSD test and two-way ANOVA were used.

## Figures and Tables

**Table 1:** Primers used in this experiment.

Name	Sequence	Note
Bulge-Loop Csu-novel-260 stem-loop primer	GTCGTATCCAGTGCAGGGTCCGAGGTATTCGCACTGGATACGACACGCCG	Reverse transcript
Bulge-Loop Csu-miR-14 stem-loop primer	GTCGTATCCAGTGCAGGGTCCGAGGTATTCGCACTGGATACGACATAGGA	Reverse transcript
Csu-novel-260-F	TTGGATGACTGGCCCATGT	qPCR
Csu-miR-14-F	GCGCGTCAGTCTTTTTCTCTC	qPCR
Csu-miRNA-universal	AGTGCAGGGTCCGAGGTATT	qPCR
*FcSDHA*-F	ACACTTTCCAGCAATGCAGGAG	qPCR
*FcSDHA*-R	TTTTCAGCCTCAAATCGGCA	qPCR
*FcDib*-F	TTCCGGAAGGCACGAATATC	qPCR
*FcDib*-R	GACTGACGAAGGGATGGATTT	qPCR
*FcEcR*-F	TGCGACAATCATCCATATACCC	qPCR
*FcEcR*-R	TCCACCTTCATTGCACACATA	qPCR
*FcSpo*-F	ATGCCAAGGAGTTGTCCTTATT	qPCR
*FcSpo*-R	CCTCGGAGAAAGTTGTCCTAATC	qPCR

## Data Availability

Not applicable.
